# CRISPR-Cas9 screens reveal common essential miRNAs in human cancer cell lines

**DOI:** 10.1186/s13073-024-01341-4

**Published:** 2024-06-17

**Authors:** Daniel J. Merk, Linda Paul, Foteini Tsiami, Helen Hohenthanner, Ghazal Mohseni Kouchesfahani, Lara A. Haeusser, Bianca Walter, Adam Brown, Nicole S. Persky, David E. Root, Ghazaleh Tabatabai

**Affiliations:** 1 Department of Neurology and Interdisciplinary Neuro-Oncology, Hertie Institute for Clinical Brain Research, University Hospital Tübingen, Eberhard Karls University Tübingen, Tübingen, 72076 Germany; 2 Genetic Perturbation Platform, Broad Institute of MIT and Harvard, Cambridge, MA 02142 USA; 3 Cluster of Excellence iFIT (EXC 2180) “Image Guided and Functionally Instructed Tumor Therapies”, Eberhard Karls University Tübingen, Tübingen, 72076 Germany; 4 German Consortium for Translational Cancer Research (DKTK), Partner Site Tübingen, German Cancer Research Center (DKFZ), Heidelberg, 69120 Germany; 5 Comprehensive Cancer Center Tübingen-Stuttgart, University Hospital Tübingen, Eberhard Karls University Tübingen, Tübingen, 72076 Germany

**Keywords:** CRISPR-Cas9 knockout, Non-coding RNA, miRNA, Common essentiality, Negative selection screens, Functional annotation

## Abstract

**Background:**

Genome-wide functional screening using the CRISPR-Cas9 system is a powerful tool to uncover tumor-specific and common genetic dependencies across cancer cell lines. Current CRISPR-Cas9 knockout libraries, however, primarily target protein-coding genes. This limits functional genomics-based investigations of miRNA function.

**Methods:**

We designed a novel CRISPR-Cas9 knockout library (lentiG-miR) of 8107 distinct sgRNAs targeting a total of 1769 human miRNAs and benchmarked its single guide RNA (sgRNA) composition, predicted on- and off-target activity, and screening performance against previous libraries. Using a total of 45 human cancer cell lines, representing 16 different tumor entities, we performed negative selection screens to identify miRNA fitness genes. Fitness miRNAs in each cell line were scored using a combination of supervised and unsupervised essentiality classifiers. Common essential miRNAs across distinct cancer cell lines were determined using the 90th percentile method. For subsequent validation, we performed knockout experiments for selected common essential miRNAs in distinct cancer cell lines and gene expression profiling.

**Results:**

We found significantly lower off-target activity for protein-coding genes and a higher miRNA gene coverage for lentiG-miR as compared to previously described miRNA-targeting libraries, while preserving high on-target activity. A minor fraction of miRNAs displayed robust depletion of targeting sgRNAs, and we observed a high level of consistency between redundant sgRNAs targeting the same miRNA gene. Across 45 human cancer cell lines, only 217 (12%) of all targeted human miRNAs scored as a fitness gene in at least one model, and fitness effects for most miRNAs were confined to small subsets of cell lines. In contrast, we identified 49 common essential miRNAs with a homogenous fitness profile across the vast majority of all cell lines. Transcriptional profiling verified highly consistent gene expression changes in response to knockout of individual common essential miRNAs across a diverse set of cancer cell lines.

**Conclusions:**

Our study presents a miRNA-targeting CRISPR-Cas9 knockout library with high gene coverage and optimized on- and off-target activities. Taking advantage of the lentiG-miR library, we define a catalogue of miRNA fitness genes in human cancer cell lines, providing the foundation for further investigation of miRNAs in human cancer.

**Supplementary Information:**

The online version contains supplementary material available at 10.1186/s13073-024-01341-4.

## Background

Noncoding elements of the human genome have attracted increasing attention. Among other classes of noncoding RNAs defined thus far, miRNAs were initially identified in nematodes [1, 2] and were later shown to form a much larger class of small RNAs, which in part are highly conserved during evolution [3]. miRNAs are transcribed as longer RNA molecules containing at least one stem-loop region which are processed to result in a mature ~ 22 nucleotide regulatory RNA molecule. These mature miRNAs can interact with the majority of human protein-coding mRNAs [4], thereby shaping the gene expression profile on the posttranscriptional level.

Much of the knowledge on cellular functions of miRNAs comes from the systematic analysis of knockout studies in several model systems. Interestingly, early analyses in *C. elegans* have suggested a minor impact of miRNAs on cellular processes, as knockout experiments have revealed abnormal phenotypes for only a small fraction of miRNA mutants [5, 6]. However, effects of miRNA knockout in flies and mice are far more severe. In flies, the vast majority of knockout mutants show a relevant phenotype including shortened lifespan or even lethality, and this was seen for both single miRNA as well as miRNA cluster mutants [7]. Phenotypes for several mouse knockout strains are similarly severe and include a wide spectrum of mostly tissue-restricted abnormalities [8–11]. Furthermore, different strategies for loss- and gain-of-function approaches including antisense oligonucleotides and mimics have been used to reveal some of the functions of miRNAs mainly in cell culture systems [12–14]. Yet, miRNA knockdown approaches using established methodologies are cumbersome and less robust than overexpression strategies.

With the advent of the CRISPR-Cas9 system, genetic loss-of-function studies have become more efficient, and this approach has become the standard method for genome-wide knockout perturbation screens. Systematic analyses of human cancer cell lines have enabled the robust identification of genes essential for cell growth and fitness in human cancer cell lines [15–18]. While these approaches were primarily intended to reveal tumor- or lineage-specific vulnerabilities, the high number of available screens has also been used to refine our knowledge of pan-essential genes which show strong depletion in the majority of cell lines during propagation. Currently available CRISPR-Cas9 knockout libraries almost exclusively target protein-coding genes and functional genomics-based investigations of the noncoding genome are relatively rare. The early genome-scale CRISPR knockout library version 2 (GeCKOv2) library contained sgRNAs targeting miRNAs [19], and a selectively miRNA-targeting CRISPR-Cas9 library has been described [20].

To facilitate the screening of miRNA function in human cells, we designed a miRNA-targeting CRISPR-Cas9 knockout library and provide a detailed comparison to previous libraries capable of targeting miRNAs. We assessed the screening performance of this library and compared the results for a screen in HeLa cells to a previous miRNA-targeting CRISPR screen in the same cell line. We then performed miRNA fitness screens in 45 human cancer cell lines, representing 16 distinct lineages, to define a set of common essential miRNAs. Our subsequent in silico network analyses and mRNA gene expression profiling allow to predict functionally relevant downstream targets of fitness miRNAs.

## Methods

### Generation of the lentiG-miR library and comparison to previous miRNA-targeting CRISPR-Cas9 libraries

The miRNA-targeting CRISPR-Cas9 knockout library lentiG-miR was generated using an algorithm from the Broad Institute [21, 22]. Throughout the manuscript, miRNA genes are referred to by their official HUGO Gene Nomenclature Committee symbol. For mature miRNAs, we follow the nomenclature from miRbase [23], omitting the species prefix as the manuscript only refers to human miRNAs (e.g., miR-483-5p and miR-483-3p). Briefly, a list of current miRNA targets was downloaded from the Ensembl BioMart in August 2020 [24], containing 1926 total unique miRNA IDs. Additionally, specific stem-loop sequences (*n* = 1917; miRbase v22) for the current set of miRNAs were obtained. Candidate Cas9-NGG PAM sequences were identified using design and scoring principles as described [21]. Valid, specific stem-loop targeting sgRNAs were preferred as candidates for the list of 1926 miRNAs, while attempting to reach a total of 5 sgRNAs per miRNA. Due to the relative length of miRNA transcripts and sequence homology, 1769 of the 1926 miRNAs were targetable with at least one valid sgRNA, with 1414 miRNAs reaching the quota of five sgRNAs. We included high specificity sgRNAs predicted to have either zero matches in the genome (non-targeting), exactly one match for common essential protein-coding genes, including spliceosomal, transcription factor, and ribosomal proteins, or random intergenic locations (one-intergenic). While miRNA stem-loop sequences served as input for the generation of all miRNA-targeting libraries investigated in this study, the design criteria used to generate these libraries differ substantially. Selection of sgRNAs in GeCKOv2 was not guided by a specific on-target efficacy prediction model but included minimization of off-target activity employing a specificity analysis (MIT score) [25]. LX-miR generation included on-target modeling (Azimuth) [21] and employed off-target assessment using the MIT score. For lentiG-miR, we used a newer release of Azimuth (Azimuth 2.0), and employed cutting frequency determination (CFD) as a model to minimize sgRNA off-target activity [21]. Comparisons across libraries targeting miRNAs were performed using Azimuth 2.0 and off-target CFD metrics. Additionally, these metrics were further used to compare functional screening performance of lentiG-miR and LX-miR libraries in HeLa cells on the basis of sgRNA depletion profiles as generated by our analysis pipeline described below.

### Cell lines

The following cell lines were used in this study and cultivated in the indicated media: D425, LN229, LNZ308, LN18, T98G, MCF7, HT1080, Huh7, HeLa, BEN-MEN, SK-N-BE, and SY5Y (DMEM supplemented with 10% v/v FCS); BT12, CHLA259, TC32, TC71, and Rh30 (IMDM supplemented with 20% FCS, 1 × ITS (insulin, transferrin, selenium)); A204, G401, HCT116, and HT29 (McCoy’s supplemented with 10% FCS); RKO and HepG2 (MEM supplemented with 20% FCS, pyruvate, GlutaMAX); CHLA06 (DMEM/F12 supplemented with B27, EGF, FGF, GlutaMAX, HEPES); MDA-MB-453 (Leibovitz’s L-15 supplemented with 10% FCS); GS-2 and GS-9 (neurobasal supplemented with B27, EGF, FGF); BT16, DAOY, Jurkat, HL60, THP1, KM-H2, JVM2, JVM3, H1048, PC9, MON, TFK1, MELJUSO, SK-Mel-30, A375, Mel1617, DU145, NCI-N87, HCC1143, and MKN45 (RPMI supplemented with 10% FCS). All cells where either authenticated prior to screening by profiling highly polymorphic short tandem repeat (STR) loci and comparison to database profiles or determined to be unique by lack of match to any STR profile. No rodent cells could be detected in any human cell line as assessed by lack of detection of mitochondrial sequences from *Mus musculus*, *Rattus norvegicus*, *Cricetulus auratus*, and *Cricetulus griseus*. Cell lines obtained from ATCC or DSMZ directly prior to screening were not authenticated. All cell lines were regularly tested for mycoplasma contamination.

### Generation of Cas9-expressing cancer cell lines

Cells were transduced with a lentivirus coding for Cas9 (Addgene #52,962) in 12well plates by spinfection in the presence of polybrene (4 μg ml^−1^). Blasticidin selection was started 24 h after transduction and kept for 5 days. After selection, Cas9 expression was verified by western blot.

### CRISPR screens using lentiG-miR

Per replicate, a total of 15 × 10^6^ cells was transduced with an appropriate volume of the lentiviral-packaged lentiG-miR library to achieve a 30% transduction efficiency (> 500 × library coverage, where coverage refers to the ratio of cell number to library size). The volume was determined for each cell line individually using a titration of the library and assessing the fraction of surviving cells after 5 days of puromycin selection as compared to cells without selection. Transductions were performed in technical triplicates. Transduction efficiency for each screen replicate was assessed using an in-line assay after 5 days of selection. Median transduction rate across all cell lines was 33%. Cells were passaged for a total of 3 weeks after viral transduction, keeping a minimum 500 × library coverage at each split. Approximately 15 × 10^6^ cells were collected, pelleted, and stored at – 80 °C at the end of the screens. Genomic DNA was extracted from the cells using QIAamp DNA Blood Midi kits. PCR amplification and Illumina sequencing were performed as previously described [21].

### Low-level CRISPR screen analysis

sgRNA library reads were demultiplexed and matched to the library reference using PoolQ (v3.3.1) [26]. Raw read counts from CRISPR-Cas9 screens can be found at figshare [27]. We determined the fraction of library-matched reads for each replicate and excluded all replicates (total of three replicates) with a fraction of matched reads of less than 60% and a total number of less than what is needed for a theoretical library average coverage of 100 reads per library construct. Sequencing of the library plasmid DNA was also performed to be used as reference and checked for sufficient sequencing depth. Furthermore, cumulative percentages of sgRNA read counts from all screen replicates were compared to the plasmid DNA. Screen replicate reproducibility was assessed using two complementary approaches. First, we calculated the Pearson correlation coefficient (PCC) between replicates after pre-filtering and excluding both common essential protein-coding controls as well as miRNAs that showed no fitness effect in any of our cancer cell lines, as suggested previously [15]. Second, we employed within-vs-between context replicate correlation scoring (WBC score) as previously described [28] both on the level of normal log_2_ fold changes (LFC) as well as differential fitness scores (dLFC) as measured by the deviation of the gene-wise consensus fitness effects.

### Correction for gene-independent effects

*CRISPRcleanR* was downloaded from GitHub [29] and used to normalize sgRNA counts for each screen replicate by scaling to the total number of reads per replicate. To run *CRISPRcleanR*, we built alignment information for the lentiG-miR and the LX-miR libraries. Each screen replicate was run separately to first correct log_2_ fold changes for gene-independent DNA cutting effects. These data were used to re-calculate corrected normalized read counts. To inspect the variation induced by the *CRISPRcleanR* correction, we both inspected sgRNA distributions of defined control sgRNA sets as well as checked for potential correction of sgRNA-level log_2_ fold changes for regions with known copy number amplification for cell lines included in GDSC1000 database [30] or elsewhere [31]. We compared this unsupervised approach to the supervised *CERES* algorithm [32] for three cell lines (HT29, A375, and TC32) with available copy number data. Ranked lists of sgRNAs based on the correction by either *CRISPRcleanR* or *CERES* were compared using a rank-biased overlap approach (RBO) [33]. RBO analyses were performed at different sgRNA depths (top 50 to 400 sgRNAs), while tuning the top weighted-ness using the value *p* of the formula so that the top 30 corrected sgRNAs contribute 92.89% to the final RBO score.

### Supervised assessment of screening performance

First, we assessed screen quality by analyzing the behavior of control sgRNAs targeting either common essential protein-coding genes or intergenic regions within the lentiG-miR library using precision-recall (PR) curves. To do that, we used the *ccr.PRRC_Curve* function within *CRISPRcleanR* yielding precision and recall in calling essential sgRNAs. Second, we applied both replicate- (null-normalized mean difference (NNMD) and Cohen’s *D*) and cell line-level quality metrics (*F* measure) to evaluate screening performance [34, 35]. We calculated the *F* measure as the harmonic mean of precision and recall (as calculated by the *pr* function of BAGEL2 [36]) at Bayesian factor five or the nearest Bayesian factor but greater than five. Direct correlation of both NNMD and Cohen’s *D* with the *F* measure revealed two outlier cell lines with poor quality (Jurkat and MDA-MB453) that were excluded from further analyses. While H1048 cells also scored at relatively poor *F* measure < 0.7, this line scored well on Cohen’s D and was included for further analysis.

### Comparison of miRNA screen data with Project Achilles

The “CRISPRGeneEffect” file from DepMap_public_23Q2 [37] was used to obtain log_2_ fold change (LFC) data for all genes from screening 1095 human cancer cell lines with the Avana library, scaled for the median LFC of internal common essential protein-coding genes to be − 1 and median LFC of known non-essential protein-coding genes to be 0. Screening data for miRNA screens were scaled similarly by the median LFC of internal common essential protein-coding genes and intergenic controls. For each cell line and screen, each gene with a LFC <  − 0.5 was defined as an essential gene, and the fraction of essential genes for each cell line was determined. To generate null distributions for both libraries, we repeated this process for either known non-essential protein-coding genes [38] (Avana) or intergenic controls binned by genomic location (lentiG-miR).

### Scoring cell line-level fitness miRNAs

Corrected sgRNA-level LFCs derived from *CRISPRcleanR* were used as input for the BAGEL2 algorithm [35] to perform a supervised gene essentiality analysis. To this end, we provided distributions of sgRNA-level LFCs for both predefined protein-coding common essentials as well as negative control sgRNAs (intergenics). Gene-level Bayesian factors were generated using the *bf* function of the BAGEL2 implementation. Corresponding recall, precision, and FDR metrics for the ranked list of Bayesian factors were calculated using the *pr* function. In addition, sgRNA-level corrected read counts derived from *CRISPRcleanR* were used as input for the MAGeCK RRA [39] algorithm to perform calling of gene essentiality using the MAGeCK Python package (version 0.5.9.5) [40]. A list of intergenic control sgRNA read counts was provided to aid in the calculation of depletion and enrichment events by permutation testing in a semi-supervised manner. Importantly, we defined *–norm-method none* in the MAGeCK test command as these counts were already normalized during the *CRISPRcleanR* process. Finally, for each cell line, we defined a list of fitness miRNAs as the overlap of genes that were considered as being essential according to both algorithms at FDR < 10%.

### Identification of common essential miRNAs

We here used the fitness percentile method to identify common essential miRNAs (CEGs) in our set of 45 human cancer cell lines screens. This unsupervised approach follows the basic intuition that if a gene is ubiquitously important for cell viability, it should fall in the top *n* depleted genes in at least 90% of all cell lines, assuming that this gene might not be top depleted in the remaining cell lines due to technical variation. Most genes will show little to no depletion in the 90th percentile of least dependent cell lines, while CEGs will still show considerable depletion in the remaining least dependent lines. We used four distinct variants (average, fixed, AUC, slope) of this method implemented in the CoRe package [41] for initial analyses. For the final set of CEGs, we used miRNA genes identified by the most stringent variant of the fitness percentile method (average). For visualization purposes, we determined non-essential miRNAs, i.e., miRNAs that do not show any fitness effect across human cancer cell lines, as the top-ranking miRNAs with the smallest LFC deviation from the median of intergenic controls.

### Generation of miRNA knockout cell lines

Selected sgRNAs from the lentiG-miR library targeting *MIR483*, *MIR663A*, or an intergenic region were cloned into the lentiCRISPRv2 plasmid (Addgene #52961) according to the resource information on Addgene. Lentiviral particles were generated in HEK293FT cells according to a standard protocol. Parental cell lines were transduced with lentiviral particles from empty lentiCRISPRv2 or the plasmid containing distinct sgRNAs using spinfection. Cells were selected for 5 days using puromycin at empirically determined concentrations. In order to rule out potential off-targeting of the here investigated sgRNAs, the following assumptions were made: in general, we hypothesized that erroneous depletion of sgRNAs targeting CEGs might be due to either (I) copy number gain of the corresponding genomic region and gene-independent cell responses due to DNA damage or (II) off-target activity resulting in knockout of protein-coding common essential genes. It is very unlikely that the majority or all of the here investigated 45 cell lines harbor a similar copy number gain at a defined genomic region, and gene-independent effects were accounted for during screen analyses (*CRISPRcleanR*, see above), thus ruling out copy number gain as cause for potentially erroneous detection of CFGs. To rule out potential off-target activity of sgRNAs for *MIR483* and *MIR663A* on protein-coding common essential genes, we determined protein-coding off-targets for these sgRNAs using CRISPRoff [42], considering all regions with up to three mismatches. Potential targeting and downstream knockout of these off-targets was investigated using transcriptomic analyses (see below), and downregulated genes were cross-referenced with the list of cancer common essential genes from the Dependency Map [17].

### miRNA network analyses

We used miRNet 2.0 to determine genes regulated by core fitness miRNAs [43]. miRBase IDs for all known mature miRNAs from common essential miRNA genes were uploaded to miRNet 2.0. Potential mRNA target genes were determined using miRTarBase v8.0 [44]. A hypergeometric test was employed to calculate the overrepresentation of functional annotations within target genes, based on the universe provided by miRNet (*n* = 16944) and the gene sets from KEGG, REACTOME, or GO databases. In order to identify significantly enriched subnetworks, we employed the *InfoMap* algorithm [45]. Functional annotation analyses for subnetwork genes were performed using a hypergeometric test.

### mRNA sequencing and downstream analyses

Total RNA from four distinct human cancer cell lines (MCF7, HT29, HL60, and PC9) for *MIR483*/*MIR663A* knockout or lentiCRISPRv2 empty control was isolated using a RNeasy Mini kit. Total RNA was subjected to library preparation using the NEBNext Ultra II Directional RNA library Prep kit. Sequencing was performed on a NovaSeq 6000 machine. Raw sequencing data has been deposited at GEO [46]. Raw sequencing data (fastq files) were analyzed using the nf-core/rnaseq pipeline v3.12.0 [47]. A detailed QC report for all sequencing runs can be found at GitHub [48]. Briefly, the pipeline included trimming of reads (Trim Galore! v0.6.4) [49], alignment to the GRCh37 genome (STAR vSTAR_2.6.1d) [50], and feature quantification (featureCounts v1.6.4) [51]. Differential gene expression analyses were performed using DESeq2 v1.38.3 [52]. Only genes with a minimum normalized count of 10 in at least 3 samples were considered for downstream analyses. Sample similarity was investigated using hierarchical clustering based on Pearson correlation coefficients or principal component analyses considering the first three components. Differentially expressed genes were calculated using pairwise comparisons of miRNA knockout cells and the corresponding empty control employing Wald statistics, considering significance at a LFC ≥ 1 and *Padj* ≤ 0.05. To test gene expression changes over several conditions at once, we employed a likelihood ratio test (LRT) considering genes at *Padj* ≤ 0.01, while regressing out gene expression changes due to differing cellular backgrounds. In order to group LRT genes by their expression changes across control, *sgMIR483*, and *sgMIR663A* cells, we performed a divisive hierarchical clustering approach using the *degPatterns* function of the DEGreport package v1.34.0 [53]. For all functional analyses, we used the clusterProfiler package v4.6.2 [54]. Predefined lists of genes were analyzed using an overrepresentation approach using the *enricher* function with the indicated gene set databases. Additionally, we performed gene set enrichment analyses based on the KEGG database using gene lists ranked LFCs from the indicated comparisons.

## Results

### Generation and validation of lentiG-miR for miRNA knockout studies

**Fig. 1:**
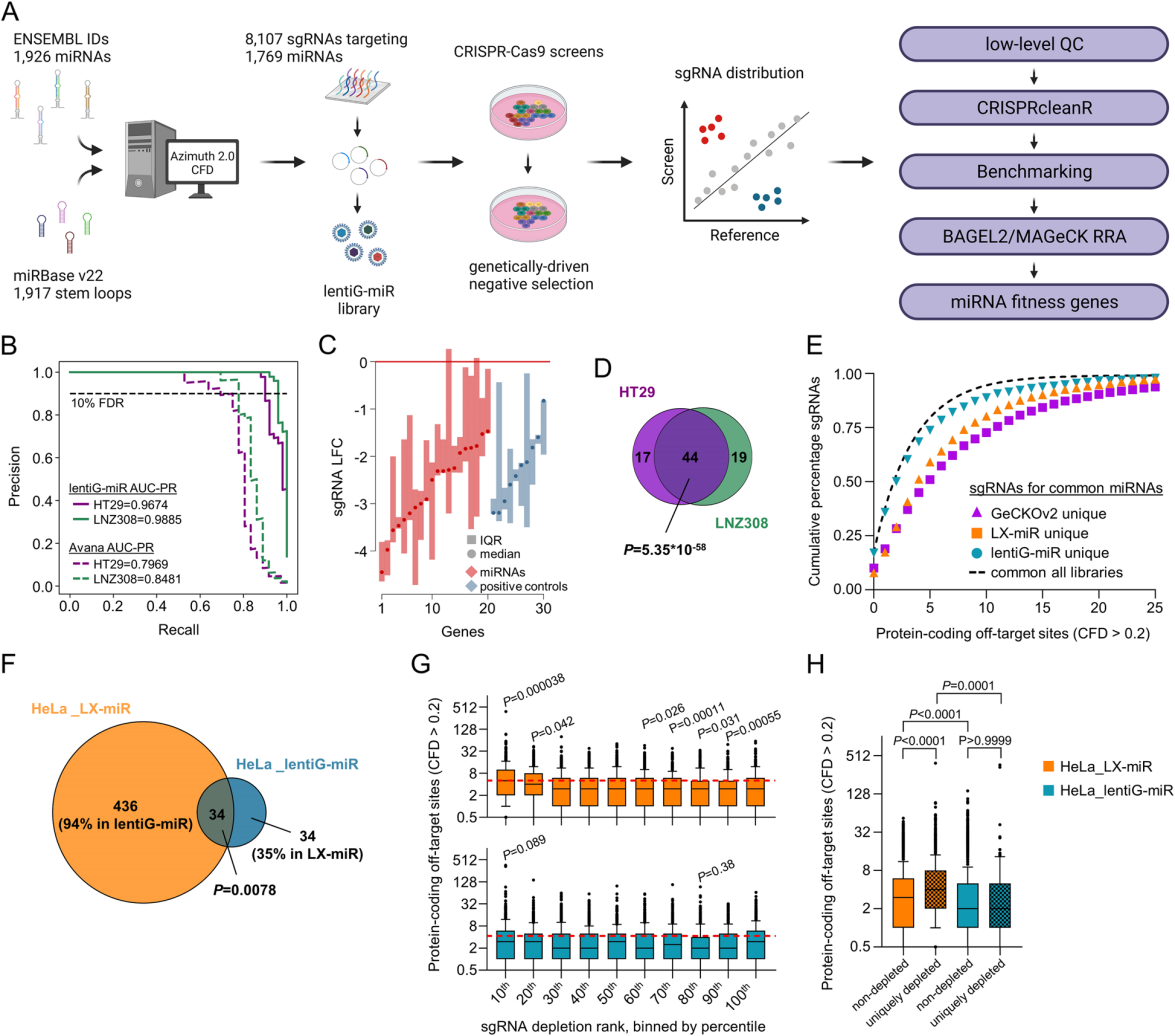
Generation and validation of a novel CRISPR-Cas9 knockout library targeting human miRNAs. **A** Schematic overview illustrating the generation of a miRNA-targeting CRISPR-Cas9 knockout library and major steps of the bioinformatic pipeline to identify miRNA fitness genes. **B** Precision-recall (PR) curve analyses to validate screening performance of the lentiG-miR library as compared to the Avana library in HT29 and LNZ308 cells. Calculated areas under the PR curve (AUC-PR) are indicated. **C** Distribution of sgRNA LFCs for the top 20 most depleted miRNAs (≥ 3 sgRNAs per gene) and common essential protein-coding genes in HT29 cells. Median and interquartile range (IQR) of sgRNA LFCs for each gene are shown. **D** Venn diagram illustrating the overlap of fitness miRNAs in HT29 and LNZ308 cells. **E** Cumulative percentage of sgRNAs targeting common miRNA genes across three CRISPR-Cas9 libraries in relation to their off-target sites. **F** Venn diagram showing the overlap of fitness miRNAs in HeLa cells as determined by the LX-miR or the lentiG-miR library. Percentages in brackets indicate coverage of respective miRNA fitness genes in the counterpart library. **G** sgRNA depletion from LX-miR (top) or lentiG-miR (bottom) libraries in HeLa cells in relation to the number of off-target sites in the human genome. sgRNAs were ranked by LFC and binned by decile. The red dashed line illustrates the mean number of protein-coding off-target sites across all bins. **H** Distribution of the number of off-target sites in protein-coding regions (CFD > 0.2) of sgRNAs targeting either non-depleted miRNAs or miRNAs determined to be essential in only the LX-miR or only the lentiG-miR screen (uniquely depleted). Data are shown as box plots with boxes showing 25th to 75th percentile and whiskers extending to the 10th and 90th percentile (**G**, **H**). Statistics are derived from a SuperExactTest (**D**, **F**), *t* tests with Holm-Bonferroni correction (**G**), and a Kruskal–Wallis test with Dunn’s correction (**H**)

We designed a CRISPR-Cas9 library targeting stem-loop sequences of miRNAs (lentiG-miR), aiming to maximize total miRNA coverage and on-target efficacy while minimizing off-target activity, with an initial quota of 5 sgRNAs per miRNA (Fig. 1A). The final library contains 8107 sgRNAs targeting 1769 human miRNAs (Additional File 1: Table S1). Not all miRNA genes had 5 or more different sgRNA site options with the minimally acceptable predicted on- and off-target profiles. In the final design, 1414, 135, 102, 71, and 47 miRNAs are targeted by five, four, three, two, and one sgRNA, respectively, so that 93% of miRNA genes were covered by at least three sgRNAs. For negative controls, we included a set of non-targeting sgRNAs that had no detected target sites in the human genome and another set of sgRNAs that each targeted only a single site within an intergenic non-coding region. For validation purposes and supervised identification of fitness miRNA genes, we also included a set of sgRNAs targeting known pan-essential protein-coding genes [16]. To assess the general screening performance of this library and to develop a suitable analysis pipeline, we first screened a small number of well-annotated cancer cell line models including HT29 and LNZ308 cells, using a library coverage of 500 cells-per-sgRNA per replicate in triplicate. Employing a pipeline provided by the *CRISPRcleanR* package [30] proved to effectively normalize sgRNA read counts and correct for gene-independent effects associated with amplified genomic regions (Additional File 2: Fig. S1 A-E). Of note, we compared the sgRNA LFC correction using this unsupervised approach to the correction employed by the supervised CERES algorithm [32] in several cell models and found that the copy number (CN) corrections were similar (Additional File 2: Fig. S1 F,G), suggesting that *CRISPRcleanR* can successfully correct for CN bias in screens performed with a low-density knockout library such as lentiG-miR.

Screens for HT29 and LNZ308 cells showed good performance in discriminating common essential protein-coding genes from intergenic controls as judged by precision-recall curve analyses, recapitulating data from the Avana library used in the Cancer Dependency Map (DepMap) [37] for these cell lines (Fig. 1B) and indicating good overall screen quality. Of note, a number of miRNAs showed robust depletion of targeting sgRNAs, and we observed a high level of consistency among redundant sgRNAs targeting these miRNAs (Fig. 1C; Additional File 2: Fig. S2 A). We next identified fitness miRNAs in each cell line using a combination of supervised Bayesian analysis of gene essentiality (BAGEL2) [35] and a semi-supervised model-based analysis of CRISPR-Cas9 knockout screens with robust ranking aggregation (MAGeCK RRA) [39] classification approaches. Defining fitness miRNAs as genes being identified at a false discovery rate (FDR) of < 10%, both algorithms showed a highly significant overlap of essentiality profiles for both cell lines (Additional File 2: Fig. S2 B). As expected in analogy to knockout screens for protein-coding genes [15], we identified both shared miRNA fitness genes as well as cell line-associated dependencies in HT29 and LNZ308 cells (Fig. 1D). Furthermore, a large proportion of targeted miRNAs are weakly expressed or unexpressed in HT29 cells [55], and the miRNA targets that scored as essential were strongly skewed towards the expressed miRNAs (Additional File 2: Fig. S2 C). Together, the same-target consistency across sgRNAs and the skew of depletion hits towards the expressed miRNAs provide evidence that screens with lentiG-miR are able to discern functionally-relevant miRNAs.

We next aimed to compare miRNA gene coverage, sgRNA composition, and screening performance of the lentiG-miR library to previously described miRNA-targeting libraries, i.e., GeCKOv2 and LX-miR [19, 20]. All libraries target highly overlapping sets of miRNA genes, with lentiG-miR targeting an additional 59 or 172 miRNAs compared to GeCKOv2 or LX-miR, respectively (Additional File 2: Fig. S3 A). However, a major difference among these libraries is the selection of sgRNA sequences used to target miRNA genes, owing to different sgRNA prediction models used during library design (see the “Methods” section for details of design criteria). Among miRNA genes covered by all libraries (*n* = 1292), 64% and 40% of sgRNAs selected for lentiG-miR are distinct from those in GeCKOv2 and LX-miR, respectively. Direct comparison of sgRNAs from these libraries using established on-target efficacy and off-target activity models [21] showed higher distributions of on-target scores for both miRNA-selective libraries as compared to GeCKOv2, with LX-miR scoring best across all three libraries (Additional File 2: Fig. S3 B). With respect to the predicted off-target activity, many potential sgRNA options were found to have better predicted off-target profiles [21] than those used in the older libraries, so that lentiG-miR by design has a superior predicted off-target profile than those previous libraries (Fig. 1E; Additional File 2: Fig. S3 C).

We next screened HeLa cells with the lentiG-miR library and directly compared the results to a previous screen in HeLa cells with the LX-miR library [20]. Of note, while some miRNA fitness genes were shared across the screens with both libraries (*n* = 34), we identified many more unique miRNA dependencies for the LX-miR library (*n* = 436) than for lentiG-miR (*n* = 34) using our bioinformatic pipeline (Fig. 1F). We next investigated a potential correlation of off-target activity and sgRNA depletion for both screens. The most strongly depleted sgRNAs in the LX-miR screen presented a significantly higher number of predicted off-targets than the less depleted sgRNAs, while we did not detect a similar correlation for the lentiG-miR library (Fig. 1G). Similarly, in the HeLa_LX-miR screen, considering only the sgRNAs that targeted hit miRNAs unique to that screen versus the HeLa_lentiG-miR screen, those sgRNAs had significantly higher predicted off-target activity than the sgRNAs that targeted the non-hit miRNAs in that screen (Fig. 1H). The distributions of predicted off-target activity in the HeLa_lentiG-miR screen were similar for sgRNAs targeting unique hits versus those targeting non-hit miRNAs. Of note, while lower on-target scores might predict less sensitivity in detecting fitness miRNAs for lentiG-miR, we did not observe a diminished competence in detecting shared fitness miRNAs in the HeLa_lentiG-miR screen as assessed by depletion of sgRNAs unique for the lentiG-miR library (Additional File 2: Fig. S3 D). Together, our data suggest lower off-target activity for sgRNAs in lentiG-miR as compared to a previously described miRNA-targeting library in negative selection screens. This, combined with the addition of 172 miRNA targets and the inclusion of a diverse set of control sgRNAs, represents advantages for the systematic investigation of miRNA function with CRISPR loss-of-function screens.

### Identification of common essential miRNAs in 45 human cancer cell lines using lentiG-miR

**Fig. 2:**
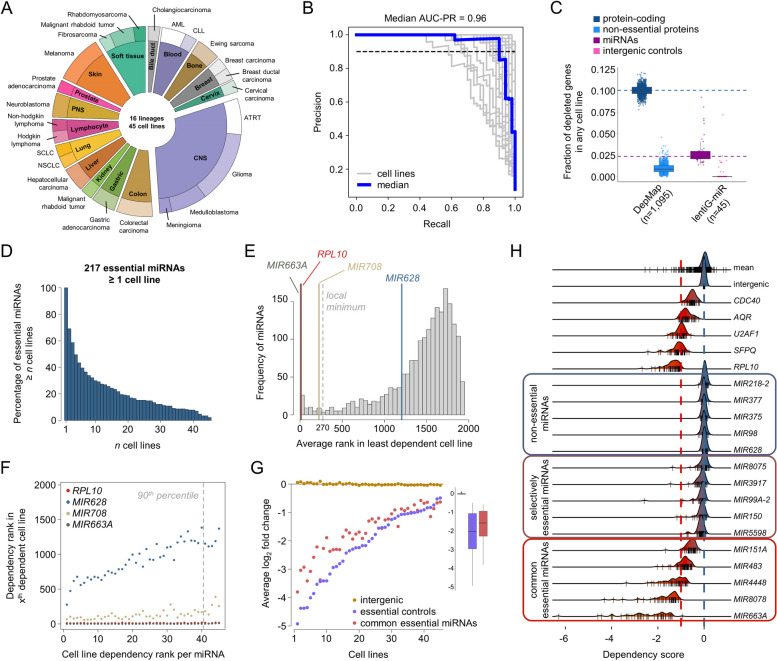
Identification of common fitness miRNAs in human cancer cell lines. **A** Donut chart illustrating all cell lines in the final analysis set grouped by lineage (inner ring) and cancer type (outer ring). **B** PR curve analyses on the basis of classifying predefined sets of essential and non-essential sgRNAs. **C** Tukey boxplot showing the fractions of depleted protein-coding/known non-essential genes in any of 1095 cell lines of the DepMap as well as depleted miRNAs/intergenic controls in any of 45 cell lines screened with lentiG-miR. Dashed horizontal lines illustrate the median fraction of depleted protein-coding/miRNA genes across the corresponding cell lines. **D** Distribution of fitness miRNAs and their number of dependent cell lines as defined by a combination of BAGEL2 and MAGeCK RRA analyses (FDR < 10%). **E** Distribution of average gene rank positions for cell lines falling at least in the 90th percentile of least dependent lines. The local minimum of a Gaussian kernel density estimate used to classify common essential miRNAs is shown. **F** Dependency ranks of exemplary genes classified as common essential (*RPL10*, *MIR708*, *MIR663A*) or non-essential (*MIR628*) and in relation to the 90th percentile. **G** Average gene-level LFCs for common essential protein-coding (essential controls) and miRNAs genes. Values for intergenic controls are shown as well. Tukey boxplot illustrates differences in median LFCs for above mentioned subsets of genes. **H** Distribution of dependency scores for selected miRNAs across 45 human cancer cell lines including means for all miRNA across all cell lines (mean), all intergenics across all cell lines (intergenic), selected common essential protein-coding genes, and selected miRNAs with the smallest LFC deviation from the median of intergenics (non-essential miRNAs) as controls. Distributions for selected selectively essential and common essential miRNAs are shown. Dashed lines indicate the median of intergenics (blue) or common essential protein-coding genes (red) across all cell lines. Vertical rug lines indicate individual cell lines

We next aimed to identify miRNAs required for cancer cell fitness across a wide range of cancer cell models, i.e., miRNAs that are common essential independent from the cellular background and tumor entity. To this end, we performed 141 CRISPR-Cas9 fitness screens in 47 human cancer cell lines, targeting 1769 miRNAs covered by the lentiG-miR library. Quality control analyses based on sgRNA read counts led to the exclusion of three replicates with insufficient coverage (< 100 × coverage). All remaining screen replicates exhibited clear changes in sgRNA abundance in all remaining replicate screens as compared to the reference plasmid accompanied by a strong depletion of common essential protein-coding genes (Additional File 2: Fig. S4 A-E). Additional replicate- and cell line-level quality metrics led to the exclusion of low-quality screens for two cell lines (Additional File 2: Fig. S4 F,G). The final data set included a total of 133 screens showing high replicate reproducibility of miRNA-targeting sgRNA effects (Additional File 2: Fig. S4 H,I), and these screens represent 45 cancer cell lines across 16 lineages (Fig. 2A). Screen data from these 45 cell lines demonstrated high precision in classifying essential and non-essential controls (Fig. 2B, and Additional File 2: Fig. S4 J). Of note, screening performance did not seem to be biased by technical factors (Additional File 2: Fig. S5).

Making use of this larger screen cohort, we first aimed to give a more detailed analysis of potentially confounding factors influencing the screening performance of lentiG-miR with regard to the genomic location of targeted miRNAs. In fact, 62% of targeted miRNAs lie within the genomic region of protein-coding genes (Additional File 2: Fig. S6 A), and we hypothesized that the overlapping host genes might influence the screening effects of those intragenic miRNAs. A direct correlation of miRNA knockout effects with their corresponding protein-coding host gene effect provided evidence for a potential bias, in particular for miRNAs that reside within exons of host genes previously classified as common essential (Additional File 2: Fig. S6 B). Further substantiating this notion, we found that a fraction of intragenic miRNAs within exons of common essential protein-coding host genes showed strong depletion across all 45 cancer cell lines (Additional File 2: Fig. S6 C,D). In order to minimize potential false positive hits, we therefore excluded all miRNAs in exons of common essential protein-coding genes (*n* = 21) from further analyses.

To next compare our miRNA screening data to knockout screens of protein-coding genes, we analyzed both DepMap data across a total of 1095 cancer cell lines as well as our miRNA screening cohort for fitness effects, considering the gene depletion effect scaled by negative and positive controls included within the respective libraries and a cutoff LFC of <  − 0.5 (see the “Methods” section). Using this metric, an average of 10% of protein-coding genes demonstrated a viability effect in any of 1095 cancer cell lines within DepMap, and 64% of all coding genes in the genome constituted a potential fitness gene in at least one cell line (Fig. 2C). In comparison, an average of only 3% of miRNA genes demonstrated a viability effect in any cell line from our data set, and only 17% of all miRNAs in the genome represented a fitness gene in at least one cell line. This may suggest that the relative frequency of fitness genes per cell line is lower for miRNAs than previously observed for protein-coding genes, but of course this difference might also reflect differences in efficacy of the CRISPR targeting of miRNA as compared to coding genes.

Taking the intersection of well-correlated BAGEL2 and MAGeCK RRA fitness calls (Additional File 2: Fig. S7 A; Additional File 3), we identified a median of 57 fitness miRNA genes in each cell line at a statistically robust threshold (FDR < 10% for both algorithms), with an average recall of 91% for essential control genes. In total, 217 (12%) of all targeted miRNAs induced a significant fitness effect in at least one cell line, and the majority of these fitness miRNAs (85%) represented a genetic dependency in fewer than 50% of all tested cell lines (Fig. 2D; Additional File 2: Fig. S7 B). We next employed the 90th percentile method to define common essential miRNAs (miRNA CEGs) across human cancer cell lines. This method relies on quantitative descriptors of fitness (i.e., rank of gene depletion in each cell line) in order to perform an unsupervised analysis, and we implemented and compared four distinct ranking criteria [41] for this approach (Additional File 2: Fig. S7 C). Using the most stringent ranking metric (average), we identified a total of 49 miRNA CEGs across 45 cancer cell lines (Fig. 2E,F; Additional File 2: Fig. S7 D,E; Additional File 4). Of those, 31 (63%) miRNAs scored as essential in more than 50% of all cell lines (Additional file 2: Fig. S7 F). Furthermore, the magnitude of depletion of miRNA CEGs was well-correlated with the magnitude of depletion of protein-coding common essential genes (Fig. 2G). Together, we conclude that a small subset of miRNAs present genetic dependencies in a highly heterogenous manner across distinct tumor cell lines. Similar to protein-coding genes [15], only a fraction of those miRNAs (i.e., miRNA CEGs) present a fitness effect in the majority or even all cell lines (Fig. 2H).

### Gene network analyses for common essential miRNAs and functional annotation

**Fig. 3:**
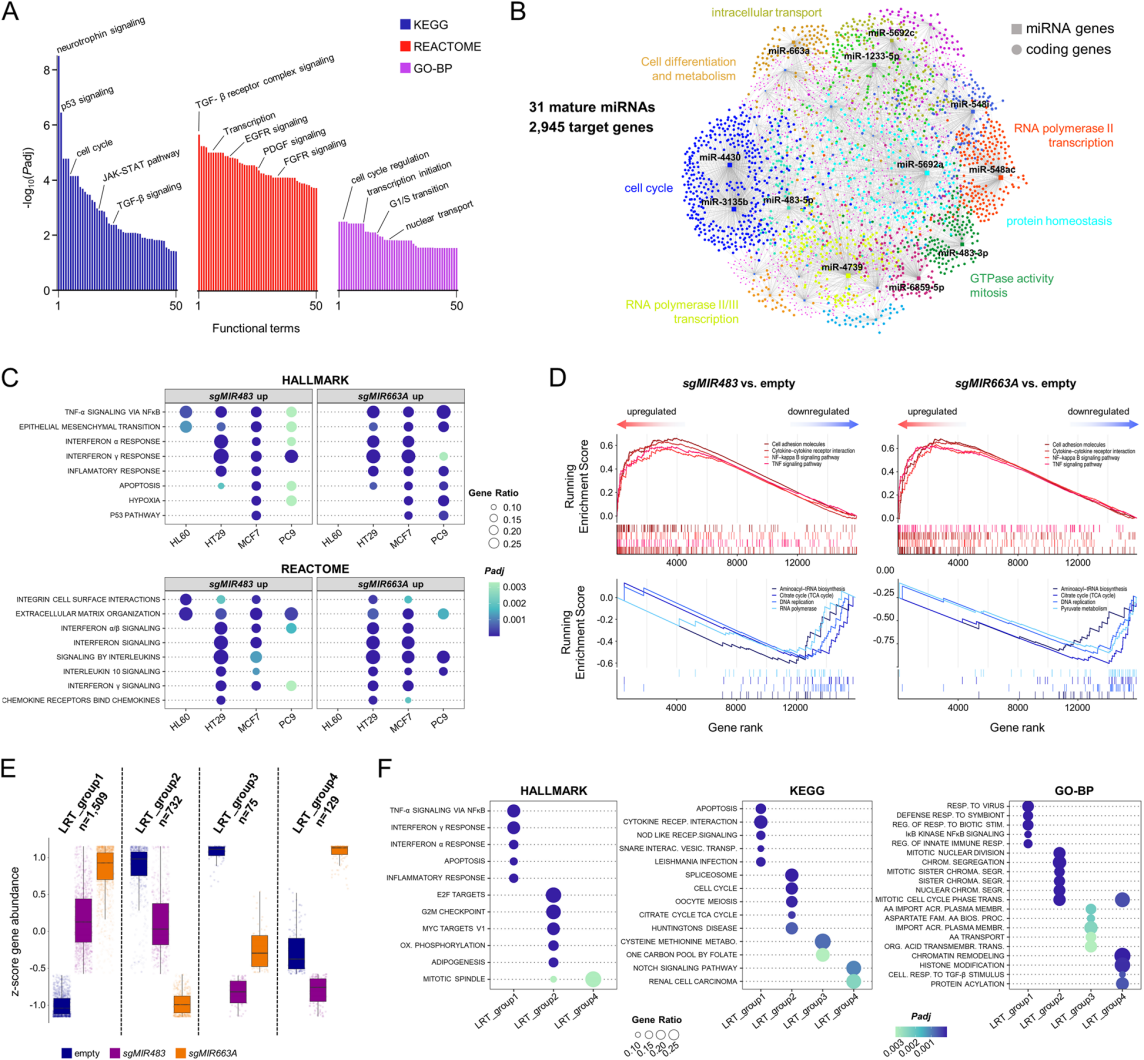
Gene network analyses for core fitness miRNAs. **A** Gene ontology analysis for all predicted protein-coding targets of common essential miRNAs using the KEGG, REACTOME, and gene ontology biological process (GO-BP) reference databases. **B** miRNA-centric interaction network for common essential miRNAs and predicted protein-coding target genes. This analysis was restricted to 31 common essential miRNAs determined to be essential in at least 50% of cell lines. Significantly enriched subnetworks were identified using the InfoMap module explorer in miRNET, and functional annotations according to GO-BP are summarized for selected subnetwork. **C** Dot plots illustrating gene ontology analyses results for genes upregulated by either loss of *MIR483* or *MIR663A* in four distinct tumor cell lines. **D** Gene set enrichment analyses using the KEGG database for the effect of loss of *MIR483* or *MIR663A* across all four cancer cell models. Selected enriched (red) or depleted (blue) gene sets for both miRNAs are shown. All gene sets score at *Padj* < 0.01. **E** A likelihood ratio test (LRT) was performed to identify genes affected by either loss of *MIR483* or *MIR663A* across all four cancer cell line models, regressing out gene expression differences due to different cell line backgrounds (cutoff *Padj* < 0.01). Genes were grouped according to their expression changes in the empty, *sgMIR483*, and *sgMIR663A* conditions using a divisive hierarchical clustering approach. **F** Dot plots showing a maximum of the top five enriched gene ontologies in distinct LRT gene groups. Statistics are derived from a hypergeometric distribution (**A**, **C**, **D**, **F**) and a likelihood ratio test (**E**). Correction for multiple testing for all statistical approaches was performed using the Benjamini–Hochberg method

Next, we set out to investigate potential gene networks regulated by miRNAs CEGs in human cancer cell lines, initially focusing on 31 miRNA CEGs which are essential in at least 50% of investigated cell lines (Additional file 2: Fig. S7 F). First, we mapped predicted miRNA-target interactions [56] for 31 distinct mature miRNAs resulting from these 31 miRNA CEGs and performed ontology analyses. Many of the resulting annotations were associated with fundamental processes such as transcription and cell cycle regulation but also included a wide array of pathways such as p53 as well as growth factor and cytokine signaling (Fig. 3A). Further network-based interrogation of miRNA-target interactions revealed a strong overlap of mRNA targets for distinct miRNAs but also showed functional enrichment in overrepresented subnetworks (Fig. 3B), indicating that subsets of miRNA CEGs act collectively to perform a biological function. However, due to the large number of predicted mRNA targets for miRNA CEGs, it does not seem surprising to identify a large number of enriched ontologies including potentially essential cellular processes, warranting further investigation of downstream effectors for individual CFGs.

We reasoned that miRNA CEGs fulfill similar functions as negative regulators of gene expression regardless of the cellular context. Thus, we analyzed in more detail the role of two selected miRNA CEGs, *MIR483* and *MIR663A*, as both of these genes have been widely recognized to act as oncogenic miRNAs [57–60]. We generated knockout clones for these two miRNAs in a total of four distinct human cancer cell lines (HL60, HT29, MCF7, PC9), representing a heterogenous group of tumor entities prevalent in humans (acute myeloid leukemia, colorectal adenocarcinoma, breast adenocarcinoma, and lung adenocarcinoma, respectively), and performed mRNA sequencing. As expected, overall differences in gene expression profiles were mainly driven by cell line identity (Additional File 2: Fig. S8 A,B). Within each cell line, miRNA knockout cells presented distinct gene expression profiles with a median of 653 and 1255 significantly deregulated genes in *MIR483* and *MIR663A* knockout cells, respectively (Additional File 2: Fig. S8 C,D; Additional file 5), with the majority of genes being upregulated by miRNA knockout (70% on average across cell lines and contrasts). Surprisingly, only a small subset of predicted mRNA targets for mature miR-483-5p/3p or miR-663a showed altered gene expression profiles, and we observed both upregulation and downregulation for these subsets of genes (Additional File 2: Fig. S8 E). Among the most consistent changes, we observed strong and robust upregulation of the cell cycle inhibitor *CDKN1A* upon loss of *MIR663A* in solid tumor cell lines, indicating that deregulation of the cell cycle might be mechanistically associated with some miRNA CEGs. Of note, expression of predicted off-targets for both sgRNAs was unchanged, ruling out erroneous targeting of potentially interfering interactors such as common essential protein-coding genes (Additional File 2: Fig. S8 F). Functionally, overrepresentation and gene set enrichment analyses revealed a common increase in cytokine signaling and apoptosis-related genes as a result of miRNA knockout, while annotations associated with active cell cycling such as transcription, translation, and energy metabolism were significantly impaired (Fig. 3C, D). Overall, we observed a highly homogenous response both across cell lines for the same miRNA knockout as well as across distinct miRNA knockout conditions (Additional File 2: Fig. S8 G), and this was particularly evident for cell lines from solid tumors which are transcriptionally more similar as compared to the leukemia cell line.

These data indicated that *MIR483* and *MIR663A* to some degree have similar target profiles or overlapping downstream mechanisms involved in maintaining cell fitness. To nominate genes commonly regulated by these two miRNAs, we performed a likelihood ratio test, controlling for gene expression differences associated with cell line identity, and performed divisive hierarchical clustering. We found four groups in total that had differing effects across our conditions, with one group (LRT_group1) composed of genes that are commonly upregulated upon loss of either *MIR483* or *MIR663A*, suggesting that these might represent direct or indirect targets of those miRNAs (Fig. 3E; Additional File 2: Fig. S8 H). A minor number of genes also showed common downregulation (LRT_group2 and LRT_group3), and only a small subset of genes had differing effects upon loss of *MIR483* or *MIR663A*. As suggested by our analyses before, genes commonly upregulated were associated primarily with cytokine signaling pathways and active apoptosis, while downregulated genes supported a mechanism where loss of either of both miRNAs leads to an impairment of cell cycle progression in human cancer cell lines (Fig. 3F). Together, our data provide evidence that miRNA CEGs are involved in the regulation of fundamental cellular processes shared across cellular contexts, and in part this might be mediated by overlapping functional properties of distinct miRNAs.

## Discussion

Here, we present lentiG-miR, a sgRNA library for CRISPR-Cas9 knockout studies of human miRNAs, and we assess its performance in comparison to previously described libraries targeting miRNA genes. We demonstrate that lentiG-miR presents advantages over older libraries in terms of on- and off-target activity metrics and overall miRNA gene coverage. We use the lentiG-miR library in a pan-cancer approach to define a set of common essential miRNAs in human cancer cell lines, advancing our insight on miRNA involvement in conserved essential processes in human cells.

Our results highlight the utility of the lentiG-miR library for effective interrogation of miRNA gene function via loss-of-function screening. Direct comparison of sgRNA metrics as well as screening performance suggests that sgRNAs from lentiG-miR have a lower degree of protein-coding off-targets and therefore likely a lower number of erroneously detected screening hits as compared to previous miRNA-targeting libraries, a difference which we attribute to improved sgRNA design criteria [21, 22]. Inclusion of targeting and non-targeting control sgRNAs represents a further advantage over previous libraries to aid in the validation of screening performance as well as the supervised detection of fitness miRNAs. At the same time, lentiG-miR retains a relatively small size which should enable screening procedures in difficult-to-grow model systems which are limited in cell number and therefore library coverage capacity. While our data suggest that *CRISPRcleanR* can effectively correct for gene-independent effects in screens performed with the low density lentiG-miR library, usage of supervised algorithms such as *CERES* might also be considered for future studies, especially for the identification of context-specific essential miRNAs. Furthermore, while lentiG-miR presents a favorable on-target to off-target balance over older libraries, further validation of the specificity and the effects of genome editing on the gene-level will be necessary on a case-by-case basis.

Making use of the lentiG-miR library, we here aimed to give a systematic functional annotation of miRNAs in the context of cellular fitness. To this end, our work provides a resource of fitness-associated miRNAs covering the vast majority of currently annotated human miRNAs. These common essential miRNAs and their corresponding target transcripts show a potential involvement of fitness miRNAs in a wide array of fundamental cellular processes such as cell cycle regulation and transcription. Additionally, our knockout studies for *MIR483* and *MIR663A* highlight a potential role of miRNA-mediated cytokine suppression as one potential mechanism contributing to cell fitness. miRNAs are known to regulate cytokines such as members of the tumor necrosis factor or interleukin family directly or by interfering with downstream pathways such as NF-κB [61, 62]. Furthermore, depending on the cellular context, it has been suggested that miRNAs act as potent cytokine repressors [63, 64]. In addition, some miRNAs might be directly involved in regulation of cell cycle progression as suggested by our finding that *MIR663A* likely inhibits the cell cycle inhibitor *CDKN1A* across distinct tumor lineages. Functionally, both *MIR483* and *MIR663A* have been shown to suppress apoptosis in cancer cells [58, 65], and this might also be related to the phenotype observed in our study. Further work will be necessary to delineate the exact mechanism of miRNA-mediated cytokine suppression, how it contributes to cellular fitness, and whether this is a phenomenon specific to cancer cells or also applies to non-malignant cells.

## Conclusions

The experimental and analytical approaches described in this study illustrate a method to uncover miRNA fitness genes in human cancer cell lines, providing insight into the integration of non-coding genes in essential cellular processes. The lentiG-miR CRISPR-Cas9 knockout library provides advantages over previous libraries with regard to miRNA gene coverage and balancing of predicted on-target and off-target activity, while providing a diverse set of high-confidence reference sgRNAs for screen validation and analysis at small library size. Thus, the lentiG-miR library should prove useful for further investigation of context-specific miRNA functions, and our set of miRNA CEGs might serve as a reference data set to filter out broadly cytotoxic candidates.

### Supplementary Information

Additional file 1: Table S1. sgRNA sequences and genomic locations for the lentiG-miR library.

Additional file 2: Figure S1. Validation of *CRISPRcleanR* for the analysis of lentiG-miR screening data. Figure S2. Validation of fitness calls from lentiG-miR. Figure S3. Comparison of miRNA-targeting CRISPR-Cas9 libraries. Figure S4. Quality control for lentiG-miR fitness screens in 47 human cancer cell lines. Figure S5. Assessment of technical confounders in miRNA CRISPR-Cas9 fitness screens. Figure S6. miRNA-host gene interaction in lentiG-miR screening data. Figure S7: Investigation of cell line-level fitness miRNAs and common essential miRNAs. Figure S8: Analysis of global gene expression changes upon loss of *MIR483* and *MIR663A* in human cancer cell lines.

Additional file 3: Table S2. BAGEL2 and MAGeCK RRA output for 47 human cancer cell lines screened with the lentiG-miR library.

Additional file 4: Table S3. Common essential miRNAs according to the 90^th^ percentile average method.

Additional file 5: Table S4. Gene expression changes upon loss of *MIR483* or *MIR663A* in HL60, HT29, MCF7, and PC9 cell lines.

## Data Availability

Bulk RNA sequencing data is publicly available at GEO (GSE242259) [46]. Original raw read counts and quality metrics from PoolQ for miRNA knockout screens can be found at figshare [27]. All major code from this study, particularly all analyses of screening data from 47 human cancer cell lines and analyses of gene expression changes upon knockout of selected miRNAs, are available on GitHub [48].

## Abbreviations

BAGEL2: Bayesian analysis of gene essentiality, version 2
CN: Copy number
CFD: Cutting frequency determination
FDR: False discovery rate
GeCKOv2: Genome-scale CRISPR knockout library, version 2
IQR: Interquartile range
LFC: Log_2_ fold change
LRT: Likelihood ratio test
MAGeCK RRA: Model-based analysis of genome-wide CRISPR-Cas9 knockout screens, robust ranking aggregation
miRNAs: MicroRNAs
miRNA CEGs: Common essential miRNA genes
NNMD: Null-normalized mean difference
PCC: Pearson correlation coefficient
WBC: Within-vs-between context replicate correlation score
RBO: Rank-biased overlap
