# Supplementary material for: CRISPR-Cas9 screens reveal common essential miRNAs in human cancer cell lines
Source: Genome Med. 2024 Jun 17;16:82. doi: 10.1186/s13073-024-01341-4 (PMC11181638; doi:10.1186/s13073-024-01341-4)
Supplement: Supplementary file 2 — Additional file 2: Figure S1. Validation of CRISPRcleanR for the analysis of lentiG-miR screening data. Figure S2. Validation of fitness calls from lentiG-miR. Figure S3. Comparison of miRNA-targeting CRISPR-Cas9 libraries. Figure S4. Quality control for lentiG-miR fitness screens in 47 human cancer cell lines. Figure S5. Assessment of technical confounders in miRNA CRISPR-Cas9 fitness screens. Figure S6. miRNA-host gene interaction in lentiG-miR screening data. Figure S7: Investigation of cell line-level fitness miRNAs and common essential miRNAs. Figure S8: Analysis of global gene expression changes upon loss of MIR483 and MIR663A in human cancer cell lines. [file 13073_2024_1341_MOESM2_ESM.pdf]

CRISPR-Cas9 screens reveal common essential miRNAs in human cancer cell lines

Daniel J. Merk, Linda Paul, Foteini Tsiami, Helen Hohenthanner, Ghazal Mohseni Kouchesfahani, Lara A. Haeusser, Bianca Walter, Adam Brown, Nicole S. Persky, David E. Root, and Ghazaleh Tabatabai

Supplementary figures

Figure S1: Validation of *CRISPRcleanR* for the analysis of lentiG-miR screening data.

Figure S2: Validation of fitness calls from lentiG-miR.

Figure S3: Comparison of miRNA-targeting CRISPR-Cas9 libraries.

Figure S4: Quality control for lentiG-miR fitness screens in 47 human cancer cell lines.

Figure S5: Assessment of technical confounders in miRNA CRISPR-Cas9 fitness screens.

Figure S6: miRNA-host gene interaction in lentiG-miR screening data.

Figure S7: Investigation of cell line-level fitness miRNAs and common essential miRNAs.

Figure S8: Analysis of global gene expression changes upon loss of *MIR483* and *MIR663A* in human cancer cell lines.

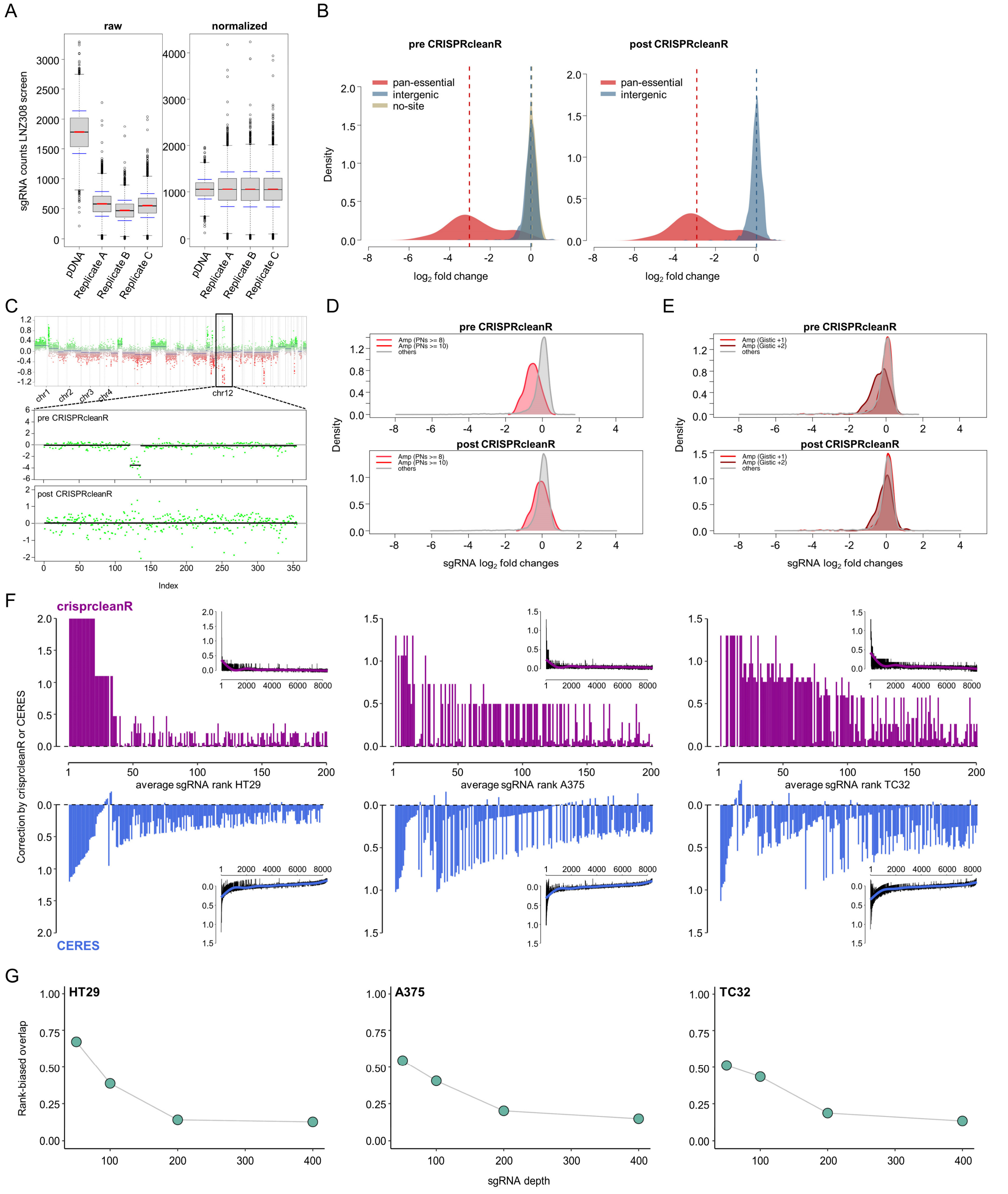

Figure S1: Validation of *CRISPRcleanR* for the analysis of lentiG-miR screening data. A Normalization of lentiG-miR sgRNA counts from plasmid DNA (pDNA) or screening replicates from LNZ308 cells by scaling individual samples by total number of reads using the *CRISPRcleanR* package. **B** Distribution of pan-essential controls, intergenic or non-targeting (no-site) sgRNA $\log_2$ fold changes in LNZ308 cells before or after correction for gene-independent effects. **C** Copy number variation plot derived from a DNA methylation array of LNZ308 cells showing copy number gain on chromosome 12 (top). sgRNA $\log_2$ fold changes on chromosome 12 of LNZ308 cells, showing a large genomic region with equal $\log_2$ fold changes before correction. **D** Density plots for sgRNA $\log_2$ fold changes in HT29 before and after CRISPRcleanR correction. sgRNAs are grouped by genomic regions known to be amplified or non-amplified according to the Genomics of Drug Sensitivity in Cancer database. **E** Same analysis as in D, but with copy number data from GISTIC2.0 analysis. **F** Correction of sgRNA-level LFCs by either *CRISPRcleanR* (top) or *CERES* (bottom) for HT29, A375, and TC32 cells. sgRNAs are ranked by their average correction for both *CRISPRcleanR* and *CERES*. The top 200 sgRNAs according to their

average correction are shown in detail. Insets show all sgRNAs in the same ranking order, with a linear regression line illustrating the trend of correction within that ranking. **G** Rank-biased overlap similarity scores are calculated at increasing sgRNA depths for ranked lists of sgRNAs from HT19, A375, and TC32 cells based on either *CRISPRcleanR* or *CERES* correction.

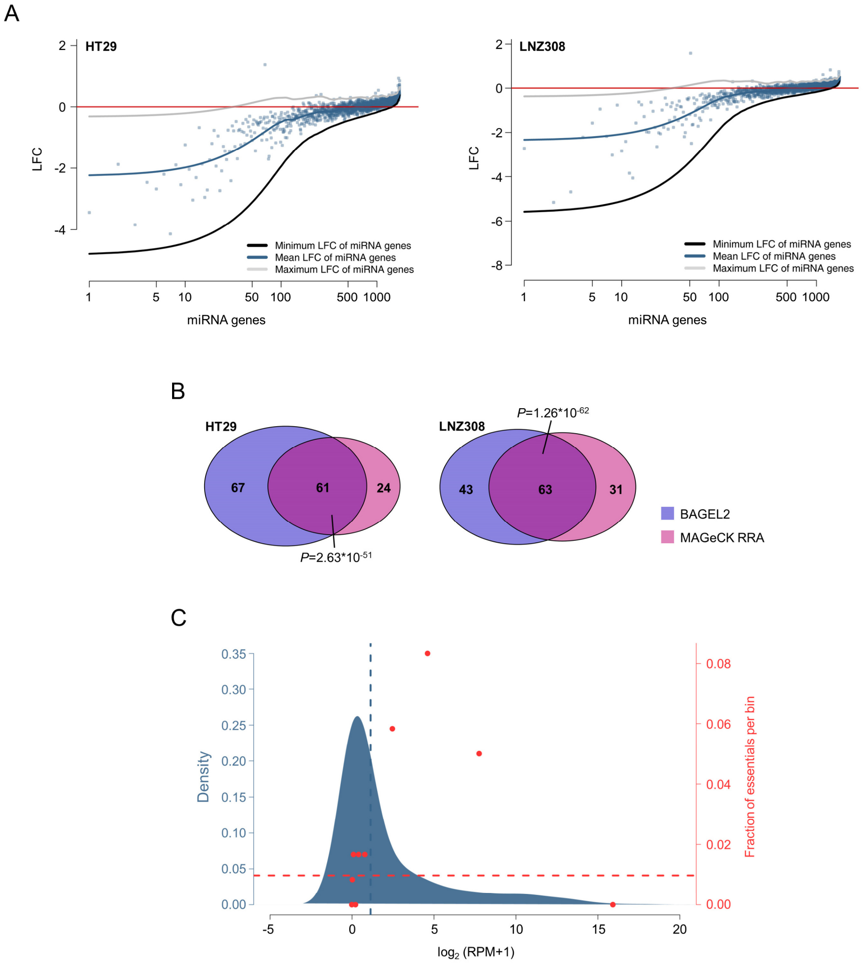

Figure S2: Validation of fitness calls from lentiG-miR. A Consistency of redundant sgRNAs across lentiG-miR screens in HT29 (left) and LNZ308 cells (right). Lines illustrate local regression lines for minimal, mean, and maximal sgRNA effects per miRNA. Points are the mean effect per miRNA. **B** Venn diagram for the overlap of classified essential miRNAs from BAGEL2 and MAGeCK RRA analyses in HT29 and LNZ308 cells. **C** Error rate estimation from gene expression in HT29 cells. miRNA gene expression density estimation in HT29 cells (blue curve, left y axis). Genes are ranked by their gene expression and binned (10 bins). For each bin, mean expression within that bin is plotted against the fraction of essential miRNAs detected at 10% FDR (overlap of BAGEL2 and MAGeCK RRA; right y axis in red). Vertical blue dashed line indicates the expression cutoff for no/trace expression of miRNAs, translating to a minimum read count of 20. Horizontal red dashed line indicates the background error rate based on the fraction of essential miRNAs detected in bins with a mean expression of less than the expression cutoff. This error rate translates to a bin-wise FDR < 20% for miRNAs with low, medium, and high expression.

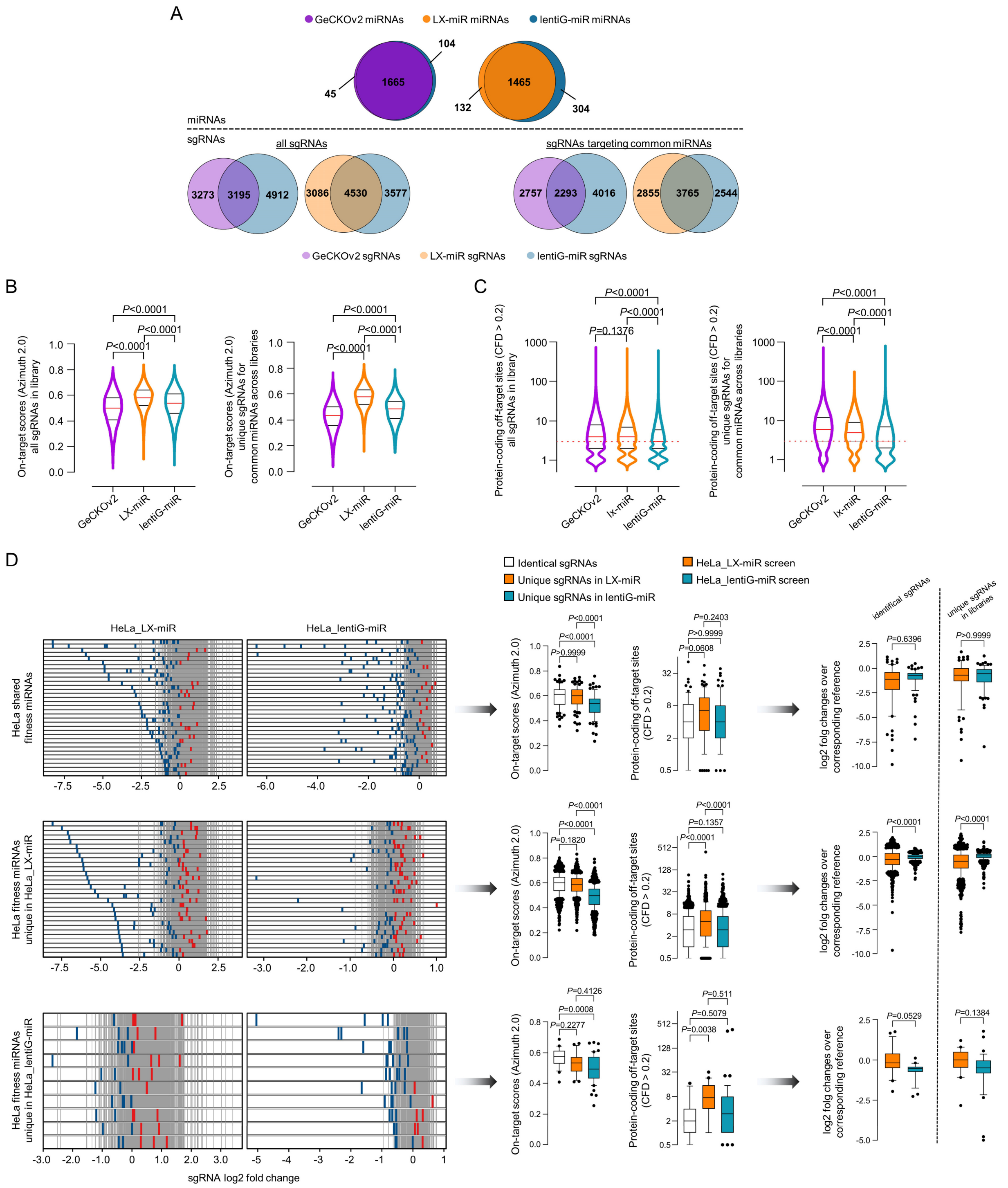

Figure S3: Comparison of miRNA-targeting CRISPR-Cas9 libraries. A Overlap of miRNA genes (top) and sgRNAs (bottom) of the lentiG-miR library with the LX-miR library and the miRNA-targeting part of the GeCKOv2 library. **B** Comparison of on-target scores for all sgRNAs (left) or unique sgRNAs targeting common miRNAs (right) in GeCKOv2, LX-miR, and lentiG-miR. **C** Comparison of the number of off-target sites in protein-coding regions with a CFD (cutting frequency determination) score > 0.2 for all sgRNAs (left) or unique sgRNAs targeting common miRNAs (right) in GeCKOv2, LX-miR, and lentiG-miR. **D** Rankview plots illustrating the distribution of sgRNA $\log_2$ fold changes for selected miRNAs depleted in HeLa cells based on screens using LX-miR or lentiG-miR (left). miRNAs are depleted according to both screens (shared fitness miRNAs, top), or present selective dependencies for individual libraries (middle, bottom). Vertical grey lines illustrate the distribution of non-targeting control sgRNAs (note the wide range of $\log_2$ fold changes for non-targeting controls in HeLa_LX-miR). miRNA genes are ordered by the magnitude of mean $\log_2$ fold changes from top to bottom for one of both screens, so horizontal lines represent identical miRNAs across paired plots for better comparison. Box plots illustrate the distribution of on-target and off-target scores for all sgRNAs from shared and library-specific fitness miRNAs (middle). For statistical comparison, sgRNAs are grouped by their overlap in LX-miR and lentiG-miR

libraries (identical sgRNAs) and their uniqueness in either of those libraries (unique sgRNAs). The magnitude of depletion for all these sgRNAs in both HeLa screens as well as their presence in only one or both libraries are shown in boxplots (right). Across all miRNA fitness genes, on-target scores for sgRNAs from lentiG-miR are lower than their respective counterparts in LX-miR. However, unique sgRNAs from the LX-miR library, in particular those targeting predicted fitness miRNAs uniquely found in the HeLa_LX-miR screen, show a significant higher number of off-target sites together with a strong depletion. Data are shown as box plots with boxes showing 25th to 75th percentile and whiskers extending to the 10th and 90th percentile. Statistics are derived from Kruskal-Wallis tests with Dunn's correction (B,C,D).

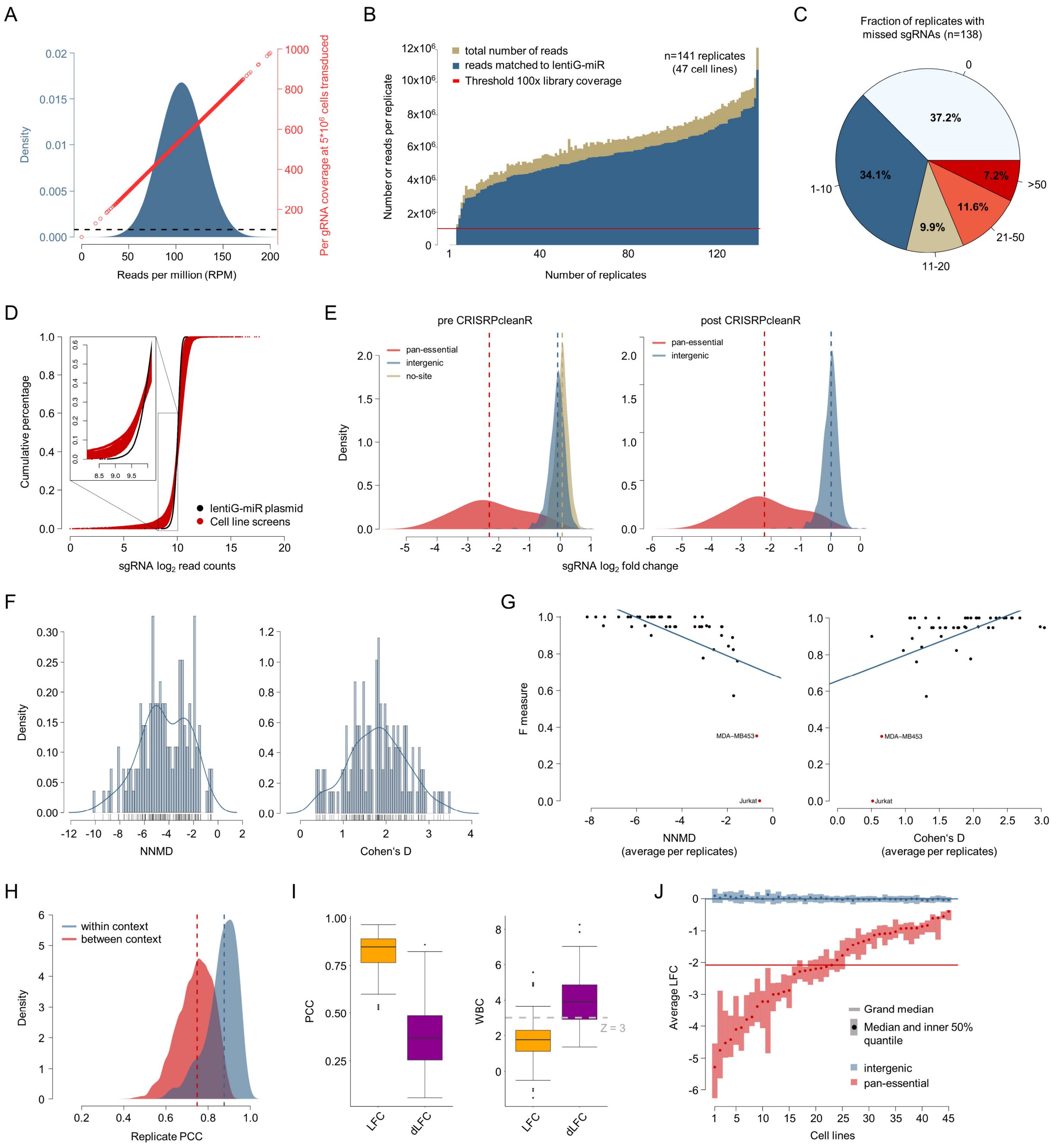

Figure S4: Quality control for lentiG-miR fitness screens in 47 human cancer cell lines. A Sequencing depth of the lentiG-miR plasmid DNA and associated sgRNA coverage for cell numbers used in CRISPR-Cas9 screening below. Dashed line indicates 100x coverage. **B** Number of total and library-matched read counts from a total of 141 screen replicates, representing 47 distinct human cancer cell lines. At a threshold of a minimum library coverage of 100x (red line), a total of three replicates from three distinct cell lines was excluded from further analysis. **C** Overview of the fraction of replicates that show missed sgRNA. **D** Cumulative percentage analysis of sgRNAs based on read counts illustrates an increase of sgRNAs with lower read counts in screening replicates as compared to the reference plasmid. **E** Distribution of reference sgRNA log_2 fold changes across all cell lines before and after correction using *CRISPRcleanR*. **F** Density distribution of null-normalized mean difference (NNMD) and Cohen's D as replicate-level quality metrics for lentiG-miR screens. **G** Correlation analysis using the harmonic mean of precision and recall at bayes factor 5 (F measure) as cell line-level quality metric, and either the average NNMD or Cohen's D across replicates of the corresponding cell line. Two cell lines, Jurkat and MDA-MB453, performed poorly in all three metrics and were excluded from further analysis. **H** Distribution of within-context and between-context Pearson correlation coefficients (PCC) scores for fitness effects (LFC) of miRNA genes essential in at least one out of 45 human cancer cell lines. **I** Between-replicate PCC (left) and within-vs-between context replicate correlation (WBC) scores (right) for LFC and dLFC data processing levels for 45 cell lines screened with lentiG-miR. **J** Median and interquartile range for gene-level LFCs for reference gene sets in screens from 45 cancer cell lines

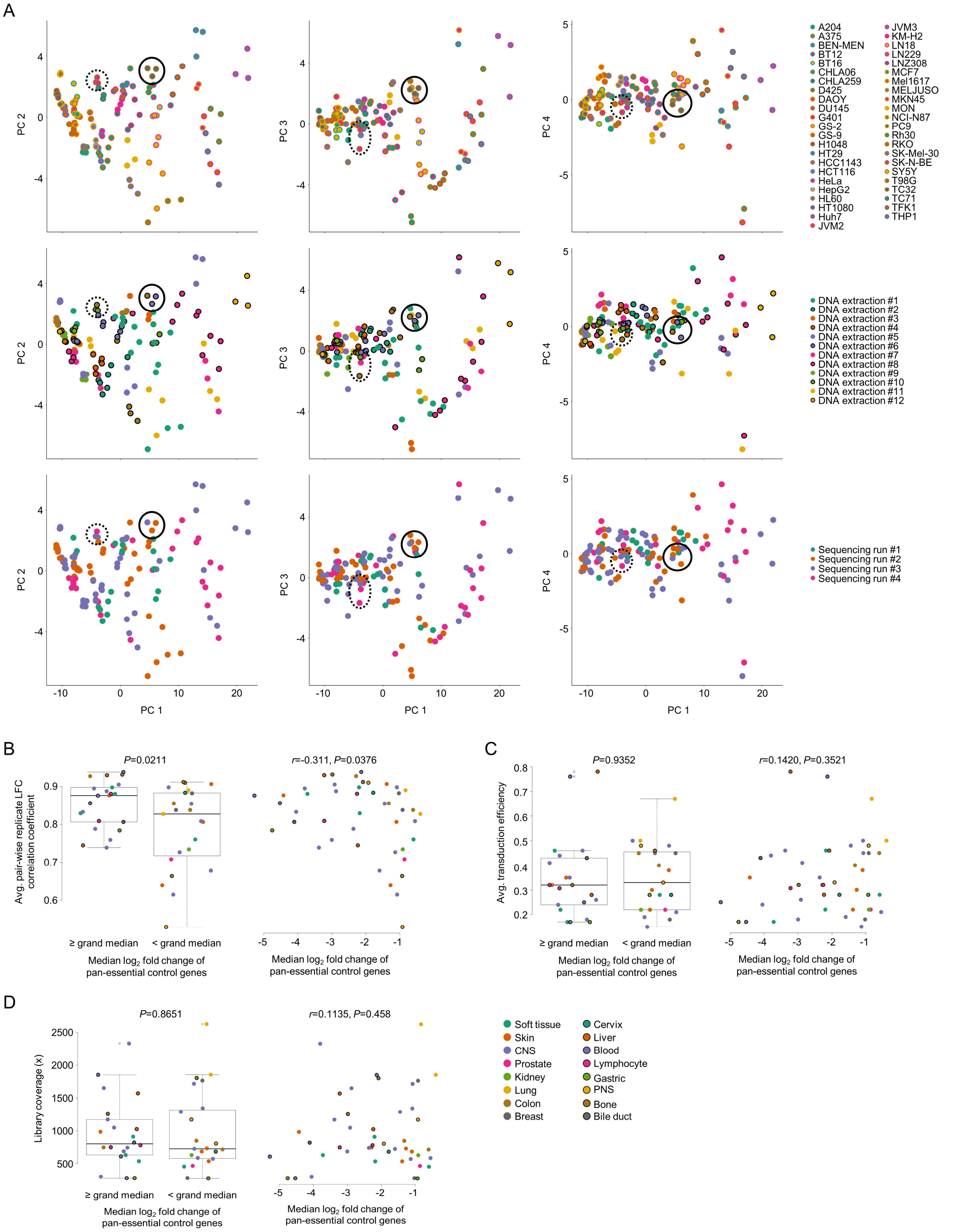

Figure S5: Assessment of technical confounders in miRNA CRISPR-Cas9 fitness screens.
A Principal component analysis (PCA) across the first four principal components for 133 high quality screen replicates from 45 human cancer cell lines based on the top 100 most variable gene-level $\log_2$ fold changes. Replicates are colored according to cell line identity (top), DNA extraction batch (middle), or sequencing run batch (bottom). Individual replicates from different cell lines cluster well across

all components. No apparent clustering according to experimental batches was seen. TC32 and SK-N-BE cell line replicates are encircled across plots as representative examples. **B-D** Cell line-level median depletion of common essential protein-coding control genes as a surrogate for screening performance and data quality. Weak correlation and significant association between replicate-level Pearson correlation coefficients and screen performance (B). No association between data quality and library transduction efficiency (C). No association between screen performance and library coverage as estimated by an in-line assay 5 days after library transduction (D). Statistics are derived from t tests (B,C,D).

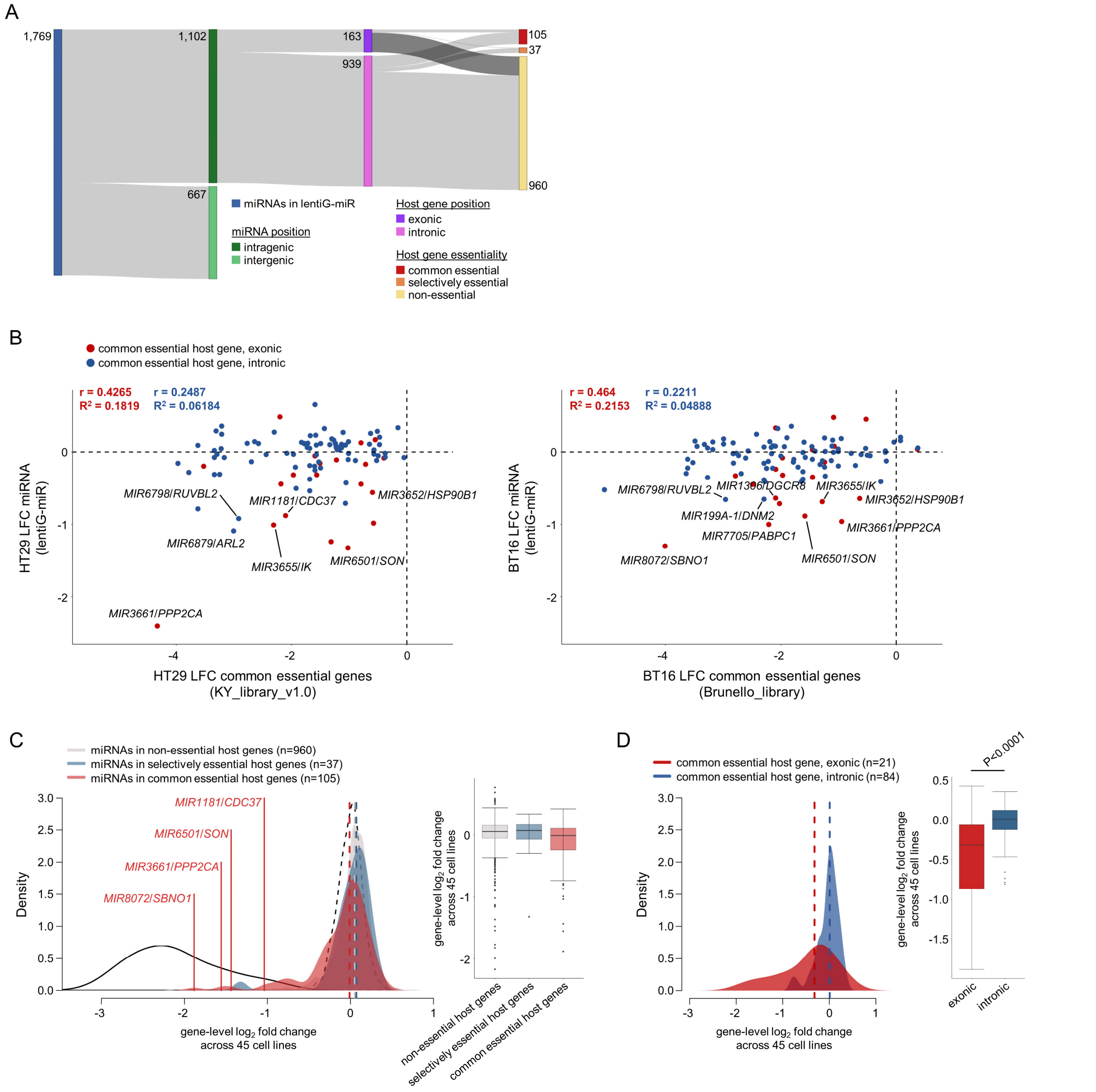

Figure S6: miRNA-host gene interaction in lentiG-miR screening data A Sankey diagram illustrating the number of intragenic and intergenic miRNAs that are targeted by the lentiG-miR library. The location of intragenic miRNAs within their corresponding protein-coding host genes and potential fitness effects of the host genes are indicated as well. Information on common or selective essentiality of protein-coding host genes was taken from the DepMap (23Q4). All other host genes were classified as non-essential, while noting that some of these genes might still be an essential gene in a restricted subset of cancer cell lines. **B** Scatter plots illustrating the correlation of fitness effects for miRNAs in common essential host genes with the fitness effect of the corresponding host gene. Protein-coding knockout screens were performed in HT29 (left) and BT16 (right) cells using the KY_library_v1.0 and Brunello libraries, respectively miRNA/host gene pairs are shown for all miRNAs determined to be essential in each cell line according to BAGEL2/MAGeCK. miRNA/host gene fitness effect correlation is shown separately for exonic and intronic miRNAs. **C** Left: Distribution of gene-level LFCs for intragenic miRNAs as determined by their average across 45 cancer cell line, grouping miRNAs by their association with non-essential, selectively essential, and common essential host genes. miRNA/host gene pairs are indicated for all miRNAs that score as an essential gene in > 50% of cell lines. Dashed vertical lines represent medians. Dashed or solid density lines represent the distribution of lentiG-miR negative and positive controls, respectively. Right: Data illustrated as boxplot. **D** Left: Same data as in C, only showing miRNAs that reside in common essential host genes, grouped by their location in exons or introns of the host gene. Dashed vertical lines represent medians. Right: Data illustrated as boxplot.

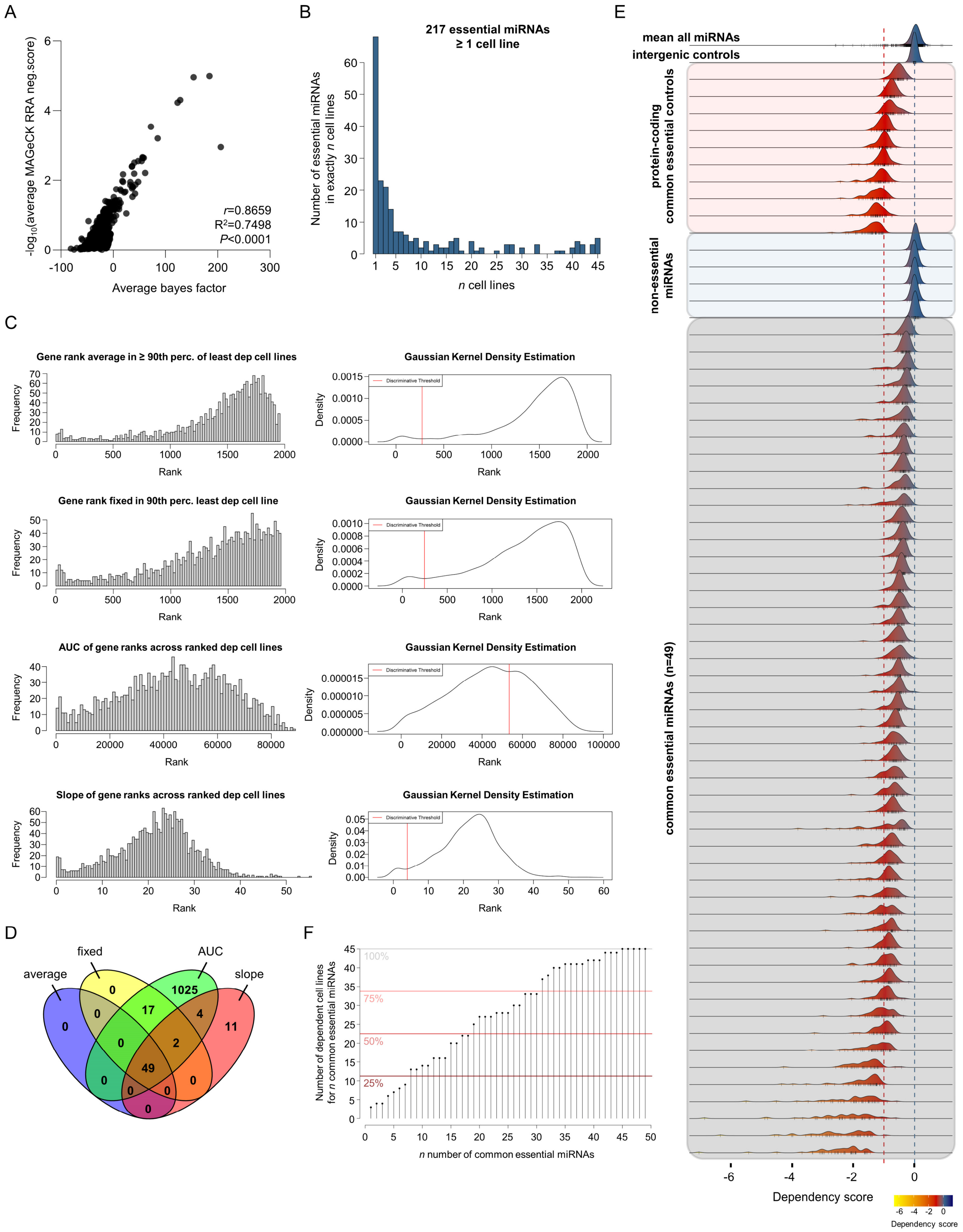

Figure S7: Investigation of cell line-level fitness miRNAs and common essential miRNAs. A Correlation analysis of average gene-level bayes factors across 45 human cell lines and average MAGeCK RRA negative scores shows high consistency across both algorithms in detecting gene essentiality. **B** Number of fitness miRNAs in a defined number of cell lines from the analysis set. **C** Gene ranks generated during the execution of the 90th percentile method according to four different criteria: average, fixed, AUC, and slope. **D** Venn diagram illustrating the differing effects of four distinct criteria (average, fixed, AUC, slope) underlying the 90th percentile

method in calling common essential miRNA genes. In the final analysis, the average method was chosen as this was the most stringent approach, encompassing only common essentials called by all other three methods as well. Note the inability of the AUC method to call biologically meaningful common essential miRNAs. **E** Ridgeline plot showing the distribution of dependency scores for all common essential miRNAs across 45 human cancer cell lines. Control distributions are the means per miRNA across all cell lines, all intergenics across all cell lines, 10 protein-coding common essential genes, or the top five miRNAs with the smallest deviation from the intergenic control median across all cell lines. Dashed lines indicate the median of intergenics (blue) or control protein-coding essentials (red) across all cell lines. Vertical rug lines for each gene indicate individual cell lines. **F** Lollipop plot illustrating the number of dependent cell lines for all 49 common essential miRNAs.

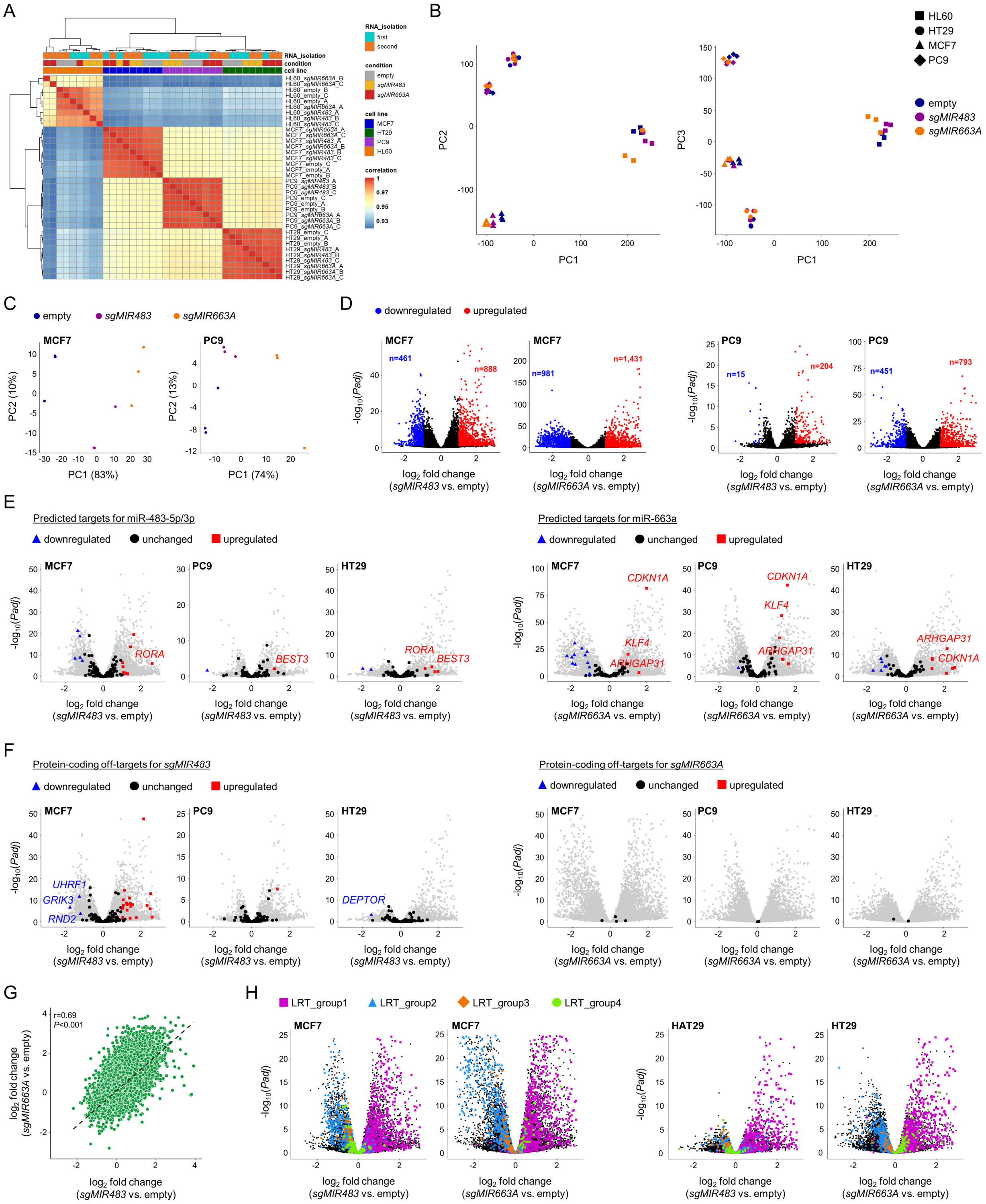

Figure S8: Analysis of global gene expression changes upon loss of MIR483 and MIR663A in human cancer cell lines. A Replicate-level pair-wise correlations for samples from four distinct cell lines (MCF7, HT29, PC9, HL60), transduced with empty, *sgMIR483*, or *sgMIR663A* lentiviral particles. **B** PCA plotting PC1 versus PC2 (left) or PC1 versus PC3 (right). Cell line identity and conditions are indicated for all 36 samples. Note the strong clustering according to cell line identity. **C** Representative PCA for two cell lines (MCF7 and PC9), performed separately for each cell line. Note the separation of samples according to condition on PC1, representing the majority of variation within the data sets. **D** Representative volcano plots illustrating gene expression changes for two cell lines (MCF7 and PC9) upon loss of either *MIR483* or *MIR663A* as compared to empty condition. Significantly depleted ($\log_2$ fold change < -1, *Padj* < 0.05) genes are indicated in blue, significantly upregulated ($\log_2$ fold change < 1, *Padj* < 0.05) genes are shown

in red. **E** Investigation of gene expression changes for predicted mRNA targets, according to miRNet, of miR-483-5p/3p (left) and miR-663a (right) upon loss of the corresponding miRNA. Selected targets for mir-483 and mir-663a and their change in expression are indicated. **F** Interrogation of predicted protein-coding off-targets, according to CRISPRoff, for sgRNAs targeting *MIR483* or *MIR663A*, considering genomic regions with up to three mismatches. The majority of gene expression levels are unchanged, and none of the few downregulated genes (indicated in blue) are classified as common essential gene as determined by the Dependency Map. **G** Correlation analysis of log_2 fold changes induced by either loss of *MIR483* or *MIR663A* across all four cancer cell lines. **H** Representative volcano plots illustrating log_2 fold changes upon loss of *MIR483* or *MIR663A* in MCF7 and PC9 cells. Genes showing consistent expression changes across all four cancer cell line models according to likelihood ratio testing (LRT_group1-4) are color-coded.
